# “Negotiorum Gestio” in Family Medicine, Informed Consent Obtainment, and Disciplinary Responsibility

**DOI:** 10.1155/2016/5767065

**Published:** 2016-03-24

**Authors:** Søren Birkeland

**Affiliations:** Department of Psychology, University of Southern Denmark, Campusvej 55, 5230 Odense M, Denmark

## Abstract

*Introduction.* Negotiorum gestio (NG) denotes an action where a person well intendedly acts on behalf of another without obtaining the latter's prior consent. In broad terms, NG-like actions have played a considerable role in health care provision. In some settings, health care delivery with only little or presumed patients' consent has been the rule rather than the exception. However, bioethical principles regarding patient autonomy and obtainment of the patient's informed consent (IC) before intervention are now increasingly materialized in the law of many countries.* Aim.* To study legal consequences of NG in family medicine and IC handling options.* Methods.* Case law examination.* Results.* A disciplinary board case is described concerning a family doctor conducting unlawful NG by not coming up to legal IC requirements.* Discussion and Conclusion.* The practical and legal implications of IC and possible role of novel Shared Decision-Making approaches in coming up to regulation and bioethical demands are discussed. It is concluded that a doctor may run an unnecessary legal risk when conducting NG in decision-competent patients and furthermore it is suggested that novel Shared Decision-Making approaches could help in obtaining a rightful and practicable IC.

## 1. Introduction

Negotiorum gestio (NG) is a legal term which, translated from Latin, means “management of business.” It denotes an action where the “gestor” as an intervenor acts on behalf, and for the intended benefit, of another person (the* dominus negotii*), however without obtaining the latter's prior consent (see, e.g., [1]). This institution may appear familiar to some medical doctors. In broad terms, the conduction of NG-like actions previously was not uncommon in health care provision. Consistent with an authoritarian (“paternalistic”) view on health care, various treatment deliveries, physical examination, and so forth were customarily performed with only little or very implicitly presumed patient consent [2].

Meanwhile, in continuation of bioethical principles of patient autonomy and self-determination, informed consent (IC) obtainment to health care intervention has been increasingly implemented internationally as both an ethical and a legal imperative [3, 4]. For example, in continuation of autonomy principles, the European Bioethics Convention (Council of Europe, 1997) maintains in its article (Article 5) that “*an intervention in the health field may only be carried out after the person concerned has given free and informed consent to it. This person shall beforehand be given appropriate information as to the purpose and nature of the intervention as well as on its consequences and risks. The person concerned may freely withdraw consent at any time.*” It is not said in Article 5 what form the IC must take (oral or written). In everyday practice, a universal claim for writing hardly would be practicable and oral IC therefore is widely accepted though signed IC would be obligatory in some (typically larger) interventions. Quite another aspect is the possible duty for doctors to record the IC in patient charts.

Similar to international recommendations, requirement for IC has been widely implemented in national regulations, and so is the case in Denmark. According to the Danish Act on Health Care Para 15, “*no treatment may be commenced or continued without the patient's informed consent* (…)” (Para 15; Act 1202, dated 14/11/2014). As a rule according to Danish law, oral IC is sufficient yet exceptions may exist (see, e.g., demand for written IC to living organ donation according to Para 52).

So far, from a legal perspective, NG in health care is a “no go.” Some remarkable exceptions however remain in formal law, that is, by way of an example, acute situations where a patient temporarily is not decision competent (the traffic victim brought in an unconscious state to the emergency ward in need of acute intervention, etc., compare Danish Act on Health Care Para 19 and “necessity”; see also, e.g., [5]). With regard to information obligations, it is stated in the Danish Act on Health Care Para 16 that “*a patient has a right to get information about his or her state of health and about treatment options including their associated risks *(…).” It is maintained as well that information must be continuously delivered, comprehensible, and individualized according to the patient's qualifications (Ministerial Order 161, dated 16/09/1998, Concerning Informed Consent Obtainment, etc.). Information should include relevant modes of treatment, alternatives, and consequences of withdrawal from treatment and it must be more comprehensive when there is a conceivable risk for serious complications or side effects.

In everyday clinical practice, the concrete assessment of whether information is required lies with the clinician. Health professionals seem to be presumed to be updated about all important information and how to distribute and communicate it to patients. As health professionals commonly seek to do their best and are required to exercise diligent conduct (cf. the Danish Act on Authorization of Health Professionals, Para 17), this arrangement at first appears reasonably straightforward. Nevertheless, dilemmas intermittently arise when it comes to the administration of (all) relevant information while reaching a sensible decision, making use of the professional know-how, and wishing to serve patients well. In order to illustrate the legal consequences of failed IC obtainment and possible solutions to the “how and how much to involve patients” problem, a tenet malpractice lawsuit concerning family medicine, judged by the Danish Health Professionals' Disciplinary Board, is described below.

## 2. Case Description

The case decision is publicly available on the Danish Health Professionals' Disciplinary Board's homepage (http://www.Patientombuddet.dk/; case number 0021330). In the following, a translated abstract is provided.

According to the case description, the patient had visited her family doctor in connection with her pregnancy. During the consultation, the family doctor “routinely” took blood samples to test for hepatitis, HIV, syphilis, and rubella. A week afterwards, the family doctor was notified by the laboratory that the HIV test was negative yet, few days later, the laboratory informed that this test actually had been inconclusive and that another one should be made. The family doctor contacted the patient by mail and asked her to have another blood test taken at the laboratory as there lacked a test result from the first test. The patient subsequently saw the family doctor to have another blood test taken. Again, a message from the laboratory said that the HIV antibody test was inconclusive. Therefore, the laboratory recommended another HIV test type which had to be repeated three months later in order to completely rule out the presence of HIV.

The complainant claimed that the defendant family doctor originally took blood samples, including an HIV test, without proper information.

It was not clear from the medical records that information had been given to the patient. The board stated that there is an obligation to obtain a patient's informed consent before the (e.g., HIV) blood sample is taken and therefore upheld that there was an obligation for the family doctor to obtain the woman's informed consent before taking the tests, including the HIV testing. Consequently, it was ruled that, during the first encounter, the litigant family doctor had violated Authorization Act obligations (cf. the legal requirement for informed consent), because she had not been sufficiently diligent in her information of the patient in connection with taking blood samples, including HIV testing. There are no facts in the case description about the final HIV test result.

## 3. Discussion

### 3.1. Tackling the IC Requirement Task and Case Interpretation

It was recently proposed that “*‘preference misdiagnosis' may be the most common form of medical error in health care*” [6]. Patients' IC often is based on the health provider's inaccurate picture of patient information, “guidance” needs, and treatment liking. The reasons may be numerous including patients' habituation to rely on doctors making health-related decisions, notions of “professionalism” that patients should be “spared” from both the tough decisions and many everyday choices, and so forth [7, 8]. Also the clinician may think that the “objectively” best option among more reasonable alternatives as well as patient preferences can be validly deduced from the clinician's medical experience. In other words, it may be tempting to lean towards the NG approach.

As it is illustrated in the case mentioned just above this innately entails some judicial aftermath: from a legal perspective, without correct information, no valid consent is obtained. So, without being legitimated by, for example, necessity, the family doctor unlawfully acted on behalf of the patient. In accordance with basic bioethical principles of patient autonomy and self-determination and legal requirements for IC obtainment, the disciplinary board concluded that this was unlawful professional conduct and issued the family doctor with a written reprimand.

Questions then arise like the following: how to ensure provision of relevant information in a way that can be taken in while respecting individual patient needs? How to protect patients from information overload? And how to help patients make the right decision without “misdiagnosing” patient desires, while simultaneously complying with IC law requirements, maintaining professionalism, and averting litigation and reprimands?

### 3.2. Shared Decision-Making (SDM) and Decision Aids (DAs) as a Potential Solution

Many health care professionals dealing with the task of information delivery and patient communication will realize this sometimes to be a difficult task. As a consequence, some clinicians may stick to the pitfall of concluding that it is most safe to provide the entire spectrum of information (all pros and particularly all the cons). Nevertheless, in many situations, this seems professionally unsatisfactory. At the same time, it is well known to most clinicians that failure to provide the information which the patient afterwards perceives as “relevant” may result in dissatisfaction, grave disappointment with health care provision, and complaints.

Consequently, there is a great variation among doctors' information practices and basically unwanted unevenness in regard to what information is provided to the concrete patient. As a possible solution to this problem, SDM approaches have been proposed as a corrective for unwanted practice deviations while also addressing patients' individual participation and information needs [9]. According to a definition of SDM, it implies “[…]* that at least two participants, the clinician and patient be involved; that both parties share information; that both parties take steps to build a consensus about the preferred treatment; and that an agreement is reached on the treatment to implement* […]” [10]. Notwithstanding SDM requires the health professional to provide all of the relevant medical facts and statistics on treatment options to patients. As “*few clinicians can quote accurate statistics from memory or eliminate their personal biases in a manner required to provide an objective presentation of the options* (…)” and furthermore sometimes may be tempted to “nudge” the patient or in any case “(…)* vary significantly on what information and risks they feel are important in making treatment decisions, which often leads them to make very different choices* (…)” [11], standardized “Decision Aids” (DAs or “decision tools,” e.g., web-based interactive programs) have been developed to assist patients and clinicians to systematically and uniformly access risk information along with highlighting patient preferences, without resorting only to memorization and physician variability. During the SDM process, patients are supported to identify individual preferences and consider features and consequences of options in order to identify informed preferences and choose the best course of action. After completing the DA it can be used for the subsequent (mandatory) patient-physician encounter. At the same time it may provide a means of documentation. SDM and DAs have further advantages of rendering a more uniform procedure of IC obtainment possible across, for example, health care sectors and borders. There has been increasing recognition of SDM and DAs as measures to enhance patient involvement in decision-making (DM), decrease decisional conflict, and improve health care communication in the substantial proportion of clinical situations where more options are reasonable alternatives [12]. With particular relevance in the present context, it should be mentioned that SDM DA is also available for use with HIV tests.

While in many countries annual numbers of malpractice lawsuits have been steadily increasing and lack of communication and information exchange seems central to patient dissatisfaction leading to malpractice litigation, it is tempting to hypothesize that SDM and DAs could serve also functions of increasing satisfaction and decreasing patients' need to complain [13–15]. Application of such measures possibly could be a step towards better compliance with bioethical principles and potentially would safeguard information provision as well as consent obtainment when embedded in the DA. Had the patient in the case described above been subject to a similar approach, it is likely that a complaint never would have evolved. If a complaint had evolved anyway, there likely would be less problem in documenting the course of IC obtainment.

## 4. Conclusions

The disciplinary decision described in this paper opposes the intermittently expressed belief among medical doctors and others that when all comes to all, patients want the doctor to decide [8]. Patients actually maintain their right to have information and to participate in health care decision-making. NG-like acts and behaviour, in addition to causing unfortunate practice variation and unpredictability for patients, put excessive responsibility on the doctor, which sometimes can be hardly honoured. In this context, attention can be drawn to the well-known Alma-Ata declaration stating that patients not only “*have the right*” but also have the “*duty to participate individually* […]* in the planning and implementation of their health care*” [16]. If NG is carried out anyway, it potentially will be “repaid with nothing but ingratitude” in a health disciplinary tribunal. In a rising number of everyday clinical situations, SDM DAs have been developed which possibly might prevent IC duty breaches in a practicable way, would provide some documentation of the IC process thereby helping doctors coming up to medical records keeping requirements, and perhaps could decrease patients' need to litigate.
